# A Review of Carbon Footprint Reduction in Construction Industry, from Design to Operation

**DOI:** 10.3390/ma14206094

**Published:** 2021-10-15

**Authors:** Banu Sizirici, Yohanna Fseha, Chung-Suk Cho, Ibrahim Yildiz, Young-Ji Byon

**Affiliations:** 1 Department of Civil Infrastructure and Environmental Engineering, Khalifa University, Abu Dhabi P.O. Box 127788, United Arab Emirates; banu.yildiz@ku.ac.ae (B.S.); 100049403@ku.ac.ae (Y.F.); youngji.byon@ku.ac.ae (Y.-J.B.); 2 Department of Chemistry, Khalifa University, Abu Dhabi P.O. Box 127788, United Arab Emirates; ibrahim.yildiz@ku.ac.ae

**Keywords:** embodied carbon, recycled asphalt, recycled aggregate, construction waste materials, alternative additives, alternative water resources

## Abstract

Construction is among the leading industries/activities contributing the largest carbon footprint. This review paper aims to promote awareness of the sources of carbon footprint in the construction industry, from design to operation and management during manufacturing, transportation, construction, operations, maintenance and management, and end-of-life deconstruction phases. In addition, it summarizes the latest studies on carbon footprint reduction strategies in different phases of construction by the use of alternative additives in building materials, improvements in design, recycling construction waste, promoting the utility of alternative water resources, and increasing efficiencies of water technologies and other building systems. It was reported that the application of alternative additives/materials or techniques/systems can reduce up to 90% of CO_2_ emissions at different stages in the construction and building operations. Therefore, this review can be beneficial at the stage of conceptualization, design, and construction to assist clients and stakeholders in selecting materials and systems; consequently, it promotes consciousness of the environmental impacts of fabrication, transportation, and operation.

## 1. Introduction

This paper aims to bring attention to the carbon footprint in the construction industry (building, maintaining, and deconstructing the structures), since the construction industry is listed as the single largest global consumer of resources [1,2]. In the European Union, building construction consumes 40% of materials and 40% of primary energy, and generates 40% of waste annually [1]. Globally, in developed and developing countries, buildings contributes to 33% of the greenhouse gas (GHG) emissions and 40% of the global energy consumption which stem from the usage of the equipment, the manufacturing of building materials and transportation [3,4]. The total CO_2_ emission of the construction sector was 5.7 billion tons which made up 23% of the emissions of global economic activity in 2009 [5]. Globally, the urban population is predicted to exceed six billion in 2045, and this could lead to more construction in the future.

According to the 4th Assessment Report of the Intergovernmental Panel on Climate Change (IPCC), GHG emissions from buildings contributed 8.6 billion t-CO_2_-e in 2004. It is predicted that it could reach up to 15.6 billion t-CO_2_-e by 2030, creating an increase of 26% CO_2_ which accounts for 30–40% of the total GHG emissions [6]. It is necessary to take action to reduce GHGs resulted from construction activities. Hence, it is vital to implement policies that focus on GHG emissions mitigation. Such schemes are broadly classified into two approaches: (1) indirect pricing such as regulations and (2) direct pricing such as carbon taxes and emission trading schemes (ETS) [7].

Regulations such as building codes can effectively reduce GHG emissions if enforced well enough, and can ensure new buildings incorporate designs that are both cost and energy effective [8]. Required codes, including the European Union’s zero energy mandate by 2021, Australia’s NatHERS 5-star standard, volunteer certificates such as Leadership in Energy and Environmental Design (LEED) which is required for all new federal government construction projects and renovations in the USA but voluntary for private construction, and the Building Research Establishment Environmental Assessment Method (BREEAM), would force designers and contractors to reconsider material usage that has a high embodied carbon content and also to rethink way they conduct their operations [9,10].

Another instrument for the mitigation of GHG emission is the carbon tax. Carbon taxes are simpler to design, have relatively low administration costs, and are attractive to stakeholders in the building sector due to their familiarity with the tax mechanism [11,12]. Carbon taxes encourage industry and the general public to help reduce GHG emissions by using energy efficiently and opting for cleaner, renewable sources of energy which in turn leads to innovations in technology and processes [13]. In terms of ETS, the cumulative amount of GHG emissions mitigated can be quantified with ETS and emission permits can be distributed for free or auctioned off [7,14]. As both energy supply and demand have equal weights, an ETS can be especially useful in the construction industry, thereby, encouraging the use of technologies that are energy efficient [15].

Studies have shown that a variety of factors slow down the move towards a carbon neutral construction industry. A study conducted in Singapore and Hong Kong found that lack of awareness, education, incentives, and high initial costs are the obstacles to such a move [16]. In another study that focused on commercial buildings in the Chinese cities of Beijing and Shanghai, the barriers were identified to be lack of regulations and financial incentives, ineffective monitoring, and lack of awareness around energy saving [17]. Therefore, this paper aims to bring attention/awareness where carbon footprint resulting from design to operation/management phases, such as manufacturing, transportation, construction, operation and maintenance, and end-of-life deconstruction in construction industry. If these sources are well identified, it will be helpful to reduce GHGs at the stage of conceptualization, design, construction, and management via selecting material, system, operation and management having less carbon footprint, which will promote environmental consciousness in whole construction operations.

There are many studies focused on CO_2_ reduction at different phases in the construction industry. However, there is no other study focusing on carbon reduction in all stages from design to operation and management phases with emphasis on manufacturing, transportation, construction, operation and maintenance, and end-of-life deconstruction comprehensively. Therefore, this paper reviewed a variety of the latest techniques for reducing the carbon footprint of each phase such as the use of alternative additives in building materials, improvements in the design, recycling of construction waste, promoting the use of alternative water resources, increasing the efficiency of water technologies, and building novel systems to improve the sustainability of the construction industry.

## 2. Carbon Footprint of Mining, Manufacturing, and Materials Transporting in the Construction Industry and GHG Reduction

**Figure 1:**
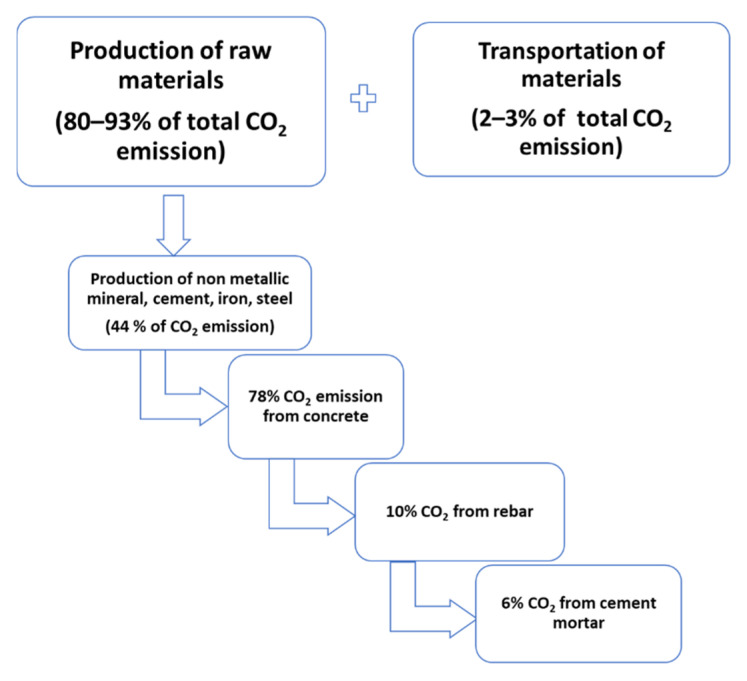
CO_2_ emission from different phases in the construction industry.

Construction process undergoes several phases, starting with production of materials (non-metallic minerals, oil, cement mortar, iron, steel, concrete) and material transportation which contributes 82–96% of the total CO_2_ emissions through the construction period as shown in Figure 1 [18,19,20,21].

A study showed that carbon footprint of urban buildings increased from 8.95 million tons in 2005 to 13.57 million tons in 2009, and that 45% of CO_2_ resulted from building material production whereas 40% of CO_2_ resulted from building energy in Xiamen, China [22]. Another study indicated that life-cycle carbon emission of a five-story brick-concrete residential building in Nanjing city of PR China was 1807.31 t, and 90% of CO_2_ were emitted at the stage of construction materials preparation and the stage of building operation [23].

### 2.1. Carbon Footprint of Limestone Quarrying

**Figure 2:**
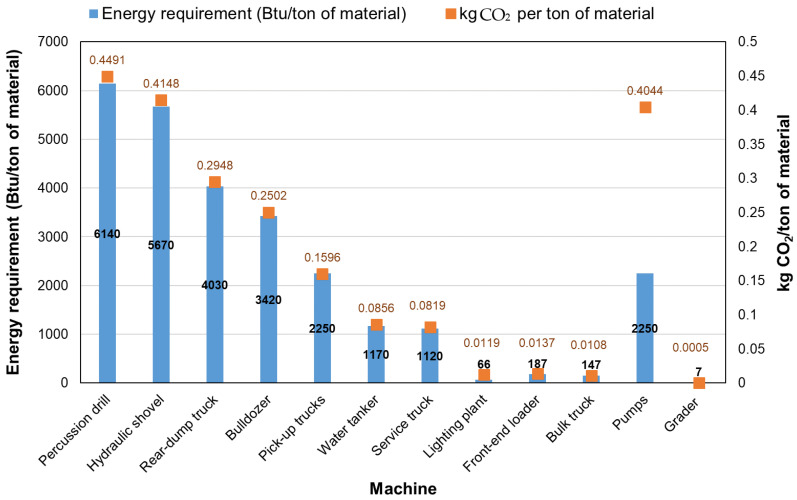
The machines used together with their energy requirements and CO_2_ emissions in limestone processing. Adapted from [24].

Limestone is one of the largest produced crushed rocks which is the basic component of construction materials, such as aggregate, lime, cement, and building stones for the construction industry [24]. The energy required for lime quarrying is associated with the machine fuel, diesel, and electricity that are needed for the limestone processing. The machines used together with their energy requirements and CO_2_ emissions are listed in Figure 2. A study found that the main cause of resource depletion in limestone quarrying was the use of diesel fuel in the transportation process, and that based on the GHG Protocol the GHGs emission was found to be 3.13 kg CO_2_ eq. per ton crushed rock product. This study suggested the adoption of alternative renewable energies such as solar, thermal, and biodiesel which will have significant impact on the reduction of GHG emissions (0.21 Mt-CO_2_ eq. annually) [25].

### 2.2. Carbon Footprint of Cement and Concrete Manufacturing

Globally, cement manufacturing accounts for 5% of CO_2_ emissions [26]. It has been reported that manufacturing of 1 kg of Portland clinker releases nearly 1 kg of CO_2_ to the atmosphere. The calcination process that takes place in the cement kiln contributes nearly 0.55 kg CO_2_ per kg of cement clinker [27]. Concrete’s typical composition is 34% sand, 12% Portland cement, 48% crushed stone, and 6% water. Since the cement percentage is relatively small in concrete, it is considered non-energy intensive compared to other construction materials [24]. CO_2_ emission rate during the production of concrete is between 347 and 351 kg of CO_2_-e/m^3^ [28]. According to Solís-Guzmán, Cement II/AL 32.5 N in two four-story blocks of flats (a total of 107 dwellings with total area of 10,243.69 m^2^) gives 148,180 kg CO_2_ eq/year and concrete HA25/B/40 gives 312,596.55 kg CO_2_ eq/year during one year of construction process [1]. A study in China reported that 1 km Portland cement concrete pavement construction gives 8215.31 CO_2_e tons in which raw material production accounts for 92.7%, concrete manufacturing phase accounts for 7.2% and onsite pavement construction phase accounts for 0.1% of the total GHG emissions [26]. The energy consumption on-site and CO_2_ emissions from the production of cement/concrete annually are listed in Table 1. The United States was the third largest producer of cement globally with 50–55 million metric tons (Mt) of CO_2_eq emissions which is equivalent to 4% of the total GHG emissions in the country in 2012. These numbers are expected to increase further as the production of cement grows [29].

### 2.3. Carbon Footprint of Asphalt Production and Construction

Asphalt is a substance used as binder for pavement materials. The energy consumption for asphalt binder production includes the extraction of crude oil, transportation, and the refining process. The energy consumption for asphalt binders is 4900 MJ per ton, and the corresponding GHG emissions is 285 kg CO_2_ per ton [30]. Heating aggregates account for 67% CO_2_ emission, asphalt heating accounts for 14% CO_2_, and mixing process accounts for 12% of total carbon emissions [31]. A case study in China reported that 20 km long asphalt pavement construction emitted 52,264,916.06 kg CO_2_-e, which includes raw materials production accounting for 43% of total GHG emissions, mixing accounting for 54% of total GHG emissions, and transportation, laying, compacting, and curing phase accounting for 3% of total GHG emission [32].

### 2.4. Carbon Footprint of Steel Production

Steel production starts with the reaction between iron ore and a reducing agent, coking coal, in the blast furnaces producing melted iron which is converted to steel in a later stage. The reaction of iron ore with carbon is the major contributor of CO_2_ emission in the steel production corresponding to 70–80% of the total CO_2_ emissions [25]. Globally, steel manufacturing accounts for 6% of CO_2_ emissions [33]. Globally, steel manufacturing accounts for 6% of CO_2_ emissions [26]. According to Solis Guzmán [1], Steel B 500S in two four-story blocks of flats (a total of 107 dwellings with total area of 10,243.69 m^2^) gives 281,898.38 kg CO_2_ eq/year during one year of construction process. Table 2 shows the energy type and consumption, and CO_2_ emissions associated with steel production in the integrated steel making and secondary steel making stages.

### 2.5. GHG Reduction in Materials and Chemicals

Alternative additives or recycled concrete waste materials can be used in common construction materials such as cement, concrete, asphalt, and clay to reduce environmental impact in the construction industry.

#### 2.5.1. Cement and Concrete Additives

Cement manufacturing requires energy; therefore, it is recommended to substitute the clinker content partially with industrial by-products. It is safe to substitute the clinker content by 30% (by weight of total binder) without compromising the strength or performance [35,36,37,38]. Also, high energy milling can be done to blend constituents to increase their reactivity and to increase their surface area, both of which can help improve the compressive strength development [39]. Recent studies have shown that regular Portland Cement can be replaced with alkali-activated slag mortars. These alkali-activated slags (AAS) can help reduce environmental impacts greatly since the production of AAS results in low energy consumption and lower energy consumption leading to lower CO_2_ emissions [40].

The admixtures used for alkali-activated slag were Peramin SRA 40 (SRA), polymer polyethylene glycol (PEG), and polypropylene glycol (PPG) [41]. In order to reduce the CO_2_ emissions, alternative clinker chemistries can be used as well as changing cement production methods in favor of more energy efficient technologies which result in reduction of 374 kg CO_2_/t clinker and totaling annual 224,540 t-CO_2_ emission release [42]. Table 3 reports CO_2_ emission reductions from some alternative technologies and materials in cement manufacturing. For instance, fluidized bed kilns can be used instead of conventional rotary kilns to burn raw materials into powder using a new technology called granulation control/hot self-granulation; such a change can lower energy consumption by 10–15% and reduce NO_x_ emissions to 0.77 kg per ton of clinker as compared to 2.1–2.6 kg per ton of clinker for conventional kilns [43]. Oxy-fuel technologies have emerged as a promising candidate for CO_2_ capture in new cement kilns by using pure oxygen for fuel burning. Due to reduced fuel combustion, oxy-fuel technology reduces CO_2_ emissions by 454–726 kg CO_2_ per ton of cement. However, due to increased electricity usage, CO_2_ emissions increase slightly by 50–68 kg of CO_2_ per ton of cement [44].

In order to mitigate the impact of concrete on the environment, its physical and mechanical properties such as strength, durability and light weight can be enhanced. For instance, lightweight concretes (LWCs) with high volume of additives such as fly ash or silica fume, which reduces the overall structural volume to withstand load, reduces CO_2_ emissions by 30–50% as compared to conventional concrete and improves mechanical properties of LWC [45]. Demolition waste such as old tires, crushed glass, and various materials from the incineration process can be granulated and cast into concrete as fillers [27]. According to a study, a sustainable Ultra High-Performance Fiber Reinforced Cement Composite (UHPFRCC) was produced using silica flour, blast-furnace slag cement, silica fume, superplasticizers, wollastonite, and steel fibers [46].

Another study stated that pulverized fuel ash (PFA) and high calcium wood ash (HCWA) were reused as concrete materials and HCWA:PFA of 50:50 and 40:60 provide the optimal flexural and compressive strength [47]. Titanium dioxide (TiO_2_) can be used as both an additive and as a coating layer. It was found that photocatalytic concrete containing TiO_2_ was effective to remove NO_x_ in urban streets [48]. According to a study, the carbon footprint of a building with 4020 m^2^ gross area and 5633 tons of total weight was 14,229 tons of CO_2_-e; in particular, this building contributed to 42% of the total emissions during both productions of material and construction stages [49]. A fractional replacement of cement in concrete with fly-ash together with the use of ground granulated blast furnace slag and the use of natural aggregates with recycle crushed aggregate can reduce up to 3.8% (10.5 kg CO_2_-e) in comparison to the conventional concrete mixture during the life cycle of the building [50].

#### 2.5.2. Asphalt Additives

Asphalt is used in most road and pavement construction, and it is a considerable contributor to GHGs in construction industry [31]. There are several additives that can assist in reducing GHG emissions, such as Sasobit, which also can reduce mixture viscosity and lower conventional mix temperature. Recent studies compared Warm Mix Asphalt (WMA) and Hot Mix Asphalt (HMA) in terms of their emission profiles. It was determined that mixture containing Sasobit additives with WMA produces the lowest CO_2_ emissions which ranges from 450 ppm to 550 ppm while HMA produces 700 ppm to 750 ppm of CO_2_ [41]. Another additive for the production of WMA is synthetic zeolite. It reduces the viscosity and increases asphalt mixtures’ workability. Furthermore, by allowing stronger coatings of bitumen on aggregates, it improves the bonding [51]. To improve the bonding of aggregates with bitumen at low temperature, zeolite can be doped with Ca (OH)_2_ which would also control the emission of CO_2_ [52]. Studies have shown that with the addition of 6% of additive by weight, mixing temperatures of asphalt mixtures reduced from 180 °C HMA to 120 °C WMA which in turn reduces the CO_2_ emissions from 7500 ppm to 500 ppm [53].

#### 2.5.3. Clay Additives

Fired and unfired clay bricks are used in the construction industry. However, fired clay bricks require a large amount of energy for their production [54]. In order to lessen the environmental impacts and achieve sustainable building industry development, unfired clay bricks are more suitable than fired bricks. Unfired clay bricks are composed of clay soils and a binder such as lime or cement [55]. Calcium-based binder such as lime and cement increases carbon in the air, due to high energy consumption during manufacturing; furthermore, the rocks naturally change CaCO_3_ into CaO which further releases CO_2_ [56]. Various additives have been tested, and it was found that MgO can be a potential alternative to calcium-based binders. MgO has some similar attributes of CaO, however MgO has the ability to immobilize heavy metals in contaminated soil. In addition, magnesite is used in manufacturing refractory products [57]. The reduction of CO_2_ emissions for unfired clay bricks were estimated 9.96 kg CO_2_-e per fu (functional unit) [50].

#### 2.5.4. Recycled Aggregate Concrete

In addition to crushed concrete, recycled aggregate concrete (RAC) consists of materials such as bricks, metals, tiles, and other materials including plastic, wood, glass, and paper [4]. RAC has inferior durability and mechanical properties as compared to conventional concrete. However, desirable RAC properties can be obtained by using admixtures such as silica fume, GGBS, fly ash, and meta-kaolin, and by modifying mixing procedures [4]. In a study, it was found that RAC together with industrial wastes such as fly ash, silica fume, blast slag, etc. can improve concrete’s carbon footprint and provide great benefits [58]. Chetumal Institute of Technology in Mexico studied the influence of the fine and normal/recycled coarse aggregates on carbon footprint [59]. The result showed that recycled coarse aggregates contributes 39% of CO_2_-e, fine aggregate contributes 19% of CO_2_-e and normal coarse aggregate contributes 42% of CO_2_-e. The study concluded that increasing recycled aggregates may help reduce 22,343 tons of CO_2_-e annually in the region of Mexico alone. A study found that 100% reclaimed and recycled aggregates, which is called Pixelcrete, reduced the content of Portland cement (60% less than the conventional concrete) in office building, and led to 73.8 t-CO_2_-e reduction in GHG emission [60].

#### 2.5.5. Recycled Asphalt

Reclaimed asphalt pavement (RAP) is used to describe re-used asphalt containing pavement. In 2010, 62.1 million tons of RAP was used in asphalt pavements [61]. The RAP could be used in three different categories of production: either as hot mix asphalt, or cold mix asphalt, or as aggregates. The RAP is generated through removal of asphalt pavement by either milling the surface using a milling machine or full depth removal. The recycling process includes both hot and cold mix asphalt and can be done in recycling plant or in place [62]. In a study, it was found that virgin asphalt produces 132 kg CO_2_-e per t. In addition, 1/3 of this value was contributed by the energy intensive processes such as heating and drying; therefore, several studies were carried out to determine the factors that affect the reduction of CO_2_ emissions including the RAP [28]. It was reported that RAP mix resulted in 5.5% reduction of carbon content, and it enhanced the reduction by 14% when larger aggregates sizes were used. By using RAP, embodied carbon content dropped to an average of 84.35 kg CO_2_-e per t [3].

## 3. Carbon Footprint of On-Site Construction Processes and GHGs Reduction

Carbon footprints are resulted during manufacturing, transportation, and installation processes of ground foundation, wood/steel/concrete framed construction at on-site construction activities. The amount of CO_2_ released from a concrete-steel residential tower in the Tehran Metropolitan City was 13,076,390,236 kg CO_2_-e, and the amount of CO_2_ emissions in 1 m^2^ of Gross Floor Area (GFA) was 435,879.67 kg CO_2_e/m^2^, of which 83% was related to the emissions from transportation of materials and 14% was related to construction wastes and 3% was related to on-site construction process [20]. A prefabricated wood-frame multi-story building in Quebec City produced a total of embodied carbon emissions of 275 kg CO_2_-e, which was 25% less than traditional buildings built with steel or concrete. The fabrication phase of building material contributed the most (75%) to the carbon emissions, while transportation (13%), construction (1%), and waste management (11%) contributed 25% [50].

A study found that the embodied carbon of a 3-bedroom semi-detached house constructed using offsite panelized timber frame was approximately 35 t-CO_2_ (82% of the total embodied carbon is embodied in the materials incorporated in the building, 2% of the total embodied carbon resulted from transportation of the materials from point of distribution to site and the rest resulted from waste materials exported from the site and energy used onsite), and an equivalent home constructed using traditional masonry construction was 52 t-CO_2_. Using modern methods in construction resulted in a 34% reduction in embodied carbon [63]. The overall CO_2_ emissions from the 1008 m wastewater pipeline project in China were calculated in tons through the entire construction period; the results were found to be 452.81 tons, 61.32 tons, and 6.59 tons from transportation phase, material manufacturing phase, and installation phase, respectively [64]. The global warming and energy consumption of 1 m^2^ of hoarding construction using large amounts of steel products and concrete in the construction site resulted in 3 tons of CO_2_ eq GWP and 39 GJ of non-renewable energy consumption [65]. Another study showed that home building with ready mix concrete results in 40% less CO_2_ emissions and less fuel consumption per lot by changing concrete slab size from 3000 ft^2^ to 1500 ft^2^. In addition, choosing the closest ready mix concrete plant saves 46 gallons of diesel and eliminates 1020 lb of CO_2_ emissions per lot in Greater Phoenix Arizona area [66].

Enhancement of energy efficiency and optimization of construction machines can reduce direct carbon emissions in construction industry [67]. Oil and electricity consumption during the on-site construction contribute to carbon footprint of construction industry. According to this study, the sources of CO_2_ emission from the on-site construction are as follows: reinforced concrete work produced 44.1 t-CO_2_ (23.9% of the total CO_2_ emissions), earthworks produced 39.1 t-CO_2_ (21.2% of the total CO_2_ emissions), ground heat construction (close loop) produced 31.9 t-CO_2_ (16.7% of the total CO_2_ emissions), foundation work (PHC PILE) produced 26.7 t-CO_2_ (14.4% of the total CO_2_ emissions), and ground heat construction (open loop) produced 16.6 t-CO_2_ (8.5% of the total CO_2_ emissions) of 84.6% of the total CO_2_ at the on-site construction phase. Furthermore, electricity consumption of concrete works on-site accounts for 41.9% of the total electricity used during the construction, resulting 14.1% (13,279 kWh) of the total electricity usage during building operations [21]. A case study has shown that on an average 99.8% of carbon present in the fossil fuel consumed by an excavator is released into the atmosphere as CO_2_ [5,67]. Additionally, emission factors during idling times contribute to overall average emission factors.

A study showed that the total CO_2_ emission increased during engine idling of non-road diesel construction equipment was considered although during the idle the time fuel use and CO_2_ emissions are between 1/3 to 1/5 of the non-idle time. During idling time, 2.7 kg CO_2_/liter was produced at a diesel fuel consumption rate of 03.7 L/h [68]. According to the EPA (2005), operators should take the equipment needs into consideration, including the time required for warm-ups and cool-downs. An operational efficiency system that is commonly accepted and used to estimate equipment productivity is 50 min = h (83%), which indicates 50 min of non-idle time and 10 min of idle time per hour. Equipment such as backhoes and bulldozers have equipment productivity ranging from 80% to 85%. However, off-road trucks have equipment productivity of 41% considering that a large part of their time is spent cycle idling, mainly loading and offloading of cargo. If off-road truck average operational efficiency increased from 40% to 50% by reducing idle time by only 6  min/h, the hourly fuel use and CO_2_ emissions can be reduced 10% [68].

A case study of a construction project in USA involved a roadway construction of an 18.8-mile highway requiring 184 pieces of machinery categorized into 35 equipment types, with idle time assumed to be 6 h per day for 7 days per week for this machinery. It was shown that the net total emission was 179,055 Mt-CO_2_-e during a period of 2.5 years (71,609 Mt-CO_2_-e per year), of which 40,023 Mt-CO_2_-e/km was contributed by the constructed roadway [69]. Amount of CO_2_ resulted from idling time can be reduced using different technologies such as direct-fire heaters, auxiliary power units (APU), thermal storage systems, on-board batteries, and automatic engine shut-off devices [70]. According to a study, direct fired heaters can reduce NO_x_ and CO_2_ emissions by 99% and 94–96%, respectively, since heat is transferred directly to the heat exchanger from the combustion flame resulting in less fuel usage than diesel engines [71].

## 4. Carbon Footprint of Construction and Demolition Waste Generation and GHG Reduction

Construction demolition waste (CDW) stems from construction, renovation, and demolition workplaces which include (i) excavation materials, (ii) road building and maintenance materials, (iii) demolition materials, and (iv) other worksite waste materials, (e.g., unpainted, non-treated wood scrap, unpainted, non-treated wood pallets, plastic, packaging), land clearing, and development activities [72]. Construction waste is increasing in volume and affecting the environment adversely [73]. Over 80% of CDW is composed of excavated earth in construction works. Mixed CDW contains the remaining of materials and packaging. [74] A 3-bedroom modular timber frame semi-detached house with 83 m^2^ internal floor area produced 17 m^3^ of waste (excavated inert materials, waste and unused construction materials, and other waste) totaling 4.9 t-CO_2_ equating to 109 kgCO_2_ per m^2^. Timber and packaging contributed to 33% and 31% of the total waste, respectively [63].

When a building reaches the end of its service life, it is demolished; the process is responsible for an emission of 0.004 to 0.01 kg CO_2_ per kg of the concrete material. This figure depends on the type of reinforcement and structure used, in addition to the general working conditions on the site during demolition [27]. A situ-concrete type building was being demolished in Korea; it required total energy consumption of 51.5 MJ/m^2^ from diesel fuel to demolish it; thus, the level of CO_2_ emitted during demolition was 10.3 kg-CO_2_/10 m^2^. In consideration of the CO_2_ that is emitted during the transportation of the demolition debris, 24.4 Kg-CO_2_/10 m^2^, 26.3 kg-CO_2_, and 17.6 kg-CO_2_ were obtained for a single-family house, a flat, and a multi-family house, respectively [75]. Waste transportation consumes energy which leads to CO_2_ emission. According to study, during the construction period, 530 tons of waste generated and during the transportation of this waste 527 L of diesel oil consumed totaling 1.4 t-CO_2_ emission from the waste transportation phase [21].

Waste materials generated from the construction industry (concrete and concrete rubble, construction ceramics, timber and wood, glass, plastics, steel, iron, aluminum, excavated soil, and Styrofoam) or from general life can be recycled as alternative construction materials [61]. During demolition, interior finishing from buildings can be reused or recycled. To look after the environment and determine the recycling and reuse values of CDW, the waste management must be planned via volume and composition determination [76]. Concrete blocks can be crushed so that they can be used for landscaping or landfilled. The fiber generated from the carpet waste can be used in fiber reinforced concrete (FCR) and fiber reinforced soil as well. The fiber improved several mechanical properties of the concrete such as toughness, strength in tension, fatigue strength, and durability, while it reduced possible cracks and defects [77]. Waste materials can act as substitutes of concrete components; it is estimated that plastic and glass can replace fine aggregates in concrete mixes by up to 20%, while waste concrete could make up for 20% of the coarse aggregate mixes in concrete [78].

Recycling one kg of aluminum as building demolition waste can contribute to emission reduction of 20.07 kg CO_2_-e [79]. Demolition debris that contains steel is separated so that the steel can be sold to scrap dealers. The economically not valuable waste can be sent to dump sites [80]. When the waste steel from hoarding construction is recycled as steel scraps, 281 kg CO_2_-e/m GHGs emission can be reduced [65]. New asphalt can be used from asphalt removed from road that is refurbished. The landscaping clearing wastes can be used as well. A portion of waste glass can be used in place of fine aggregate in asphalt paving mixtures (glassphalt) [81]. Reusing wood waste in production of particleboard reduced embodied carbon emissions up to 14.6% (−28.6 kg CO_2_-e/m^2^) [50].

## 5. Carbon Footprint during Operational Stage and GHGs Reduction

Over the full cycle, building operations contribute to the CO_2_ balance when in service [82]. Carbon emission during operational stage of a building was a major contributor, accounting for 85.4% of the total emission followed by the construction stage which accounted for 12.6% of total emissions [83]. A high-rise residential housing block in Hong Kong demonstrated that GHG emission was estimated to about 213.03 t-CO_2_-e/flat and 4980 kg CO_2_-e/m^2^, of which 85.82% was stemming from the operating energy, 12.69% from materials, 1.14% from renovation, 0.28% from end-of-life of the building, and 0.07% from other factors [84]. The energy consumption per area of the buildings from urban, national, and global scales are 3.03 GJ/m^2^, 4.27 GJ/m^2^ and 0.44 GJ/m^2^ which correspond to 0.40 t-CO_2_-e/m^2^, 0.14 t-CO_2_-e/m^2^ and 0.04 t-CO_2_-e/m^2^ greenhouse gas emissions, respectively, based on hybrid systems analysis combining input–output analysis and process analysis in China [85].

In order to contribute to CO_2_ reduction, new technologies were implemented in buildings. According to a study, low-carbon strategies, such as increased energy efficiency design for new buildings and energy-saving retrofit for existing buildings would decrease energy consumption by 2.98% with a carbon emission reduction of 3.15 million t-CO_2_-e [22]. Choosing correct materials, systems, and technologies which are listed in following sections at the phase of design and materials selection, will reduce energy consumption and CO_2_ emissions during operational stage of the buildings.

### 5.1. Alternate Water Resources for Water Reuses

Reusing water in a typical office building is estimated to conserve about 75% of the indoor potable water [86]. The average water saving of a green building was estimated to reach 37.6% with applying water efficiency technologies [87]. The rise of the water savings will reduce energy consumption and CO_2_ emissions [88]. The passive irrigation system has two stages: collecting water when it rains and supplying water in drought conditions [89]. Water flow in the system is natural under gravity or capillarization method [90]. A 250-room hotel in Birmingham, UK, with the rainwater recycling system saved up to 780 m^3^ of potable water per year [91]. According a comparative simulation model, gravity fed rainwater harvesting system for a high rise building in Mexico saved up to 8.5% of GHG [92]. Graywater is the water produced by bathroom, laundry machines, sinks, showers, and bathtubs [93]. Treated graywater can be reused for landscape irrigation and toilets [86].

Efficiency of water use can be improved by graywater recycling systems for flushing of toilets by dual piping, which will contribute to reducing urban water demand from 10% to 25% [94]. NH Campo de Gibraltar hotel substitutes 20% of potable water with filtered and treated grey water from showers, which resulted in a 20% reduction in annual water bill [91]. Blackwater comes from toilets and kitchens. Blackwater reuse showed a positive response from people who used automated or remotely controlled systems by the installer. Another study reported that it is costly and has poor process design [95]. Condensate recovery reuses water produced by air conditioning (AC) systems [95]. AC condensate can be used in flushing toilets, irrigation, cooling towers, roof cleaning, green roofs, and spray cooling [96].

Examples of water reuse and alternative water supplies include water conserving toilets, waterless toilets, waterless urinals, alternative shower and faucet fixtures (alternative controls, self-powering, low flow), water efficient appliances, and alternative landscaping (high efficiency irrigation, water conserving plant selection) [95]. Some statistical studies showed that water technologies increase water efficiency. For instance, urinals and commercial dishwashers showed the greatest reductions of water use, while showers and commercial toilets showed the least savings [88]. In the same manner, wastewater centralized reuse system (WWCRS) require more energy for treatment which leads to higher CO_2_ emissions, while the greywater decentralized reuse system (GWDRS) requires less energy (11.8–37.5%) than WWCRS consumed [97]. A constructed wetlands system treats wastewater in a building so that it can be used in low-flow toilets and urinals, which reduces the water use in total by a percentage higher than 60% [86].

### 5.2. Heating, Ventilation and Air Conditioning

Heating, ventilation, and air conditioning (HVAC) systems of buildings consume about 40–60% of total energy taking into consideration the embodied energy which stems from the production of the building [98]. Owing to their large thermal mass, concrete and other heavy weight materials positively impact the energy consumption of buildings; for a heavy weight building (based on concrete frame), energy needed for heating/cooling/ventilation is 10 MJ/m^2^ resulting in 1.3 CO_2_/m^2^; and for light-weight building, (based on plaster boards stud walls), it is 20 MJ/m^2^ resulting in 2.6 CO_2_/m^2^ in Northern Europe [27].

Equipment sizing and selection, pipe/duct sizing, energy performance analysis, system optimization, real-time performance optimization, control analysis, control optimization, and simulation and programming for HVAC systems can reduce energy consumption and increase the comfort of residents [99,100]. According to a study, using a high energy performance air conditioner resulted in 7664.4 t-CO_2_-e reduction in an office building in Nanhaiyiki 3, China; 451.5 t-CO_2_-e reduction in a Pixel building in Australia during the life cycle of the buildings. In the same fashion, using natural ventilation and lighting resulted in a 5687.6 t-CO_2_-e reduction in Nanhaiyiki 3, China; 4649.8 t-CO_2_-e reduction in the Pixel building in Australia during the life cycle of the buildings [60].

### 5.3. Other Building Systems and Technologies

There are various technologies and systems that can be applied to enhance the efficiency of buildings and decrease CO_2_ emissions. Such innovations include: windows and building surfaces with tunable optical properties; high-efficiency heat pumps; highly efficient lighting devices; thin insulating materials; improved software for analyzing building design and operations; inexpensive, energy harvesting sensors and controls; optimized control strategies; and interoperable building communication systems [101]. A study was conducted to compare different systems in a building, and it found that systems like interior lights (−150%), mechanical ventilation (−25%), and pumps (−11%) had the least energy savings whereas systems like interior fans (100%), heat rejection units (56%) and receptacle equipment (33%) had the highest energy savings. The negative values show that the systems are less efficient when compared to the baseline [82]. In another study, it was found that using renewable energies such as a solar photovoltaic system, wind turbine, and anaerobic digester resulted in 1204.1 t-CO_2_-e reduction in an office building in Australia, and using renewable energy such as a solar photovoltaic system, a solar thermal water system, and a ground source heat pump resulted in 2871.6 t-CO_2_-e reduction in an office building in China during the life cycle of the buildings [60]. Expanded polystyrene (EPS), cellulose, and elastomer as insulation and sealing materials resulted in an average 3.5 kg CO_2_-e/kg emission, some insulation materials such as sheep’s wool could reduce its impact up to 98% [50].

## 6. Discussion

Globally, in the developed and developing countries, the whole process of construction and building operations contributes to 33% of greenhouse gas (GHG) emissions and 40% of global energy consumption, stemming from the usage of the equipment, transportation, and the manufacturing of building materials. The urban population is increasing, which leads to more construction in the future and increased GHGs emissions [6]. Therefore, new policies are required for mitigation of GHG emissions. Regulations such as building codes can effectively reduce GHG emissions if enforced well enough and can ensure new buildings incorporate designs that are both cost and energy effective. However, regulations alone can result in extra costs for the governments, and they should be designed to cover all aspects of GHG emission activities [7]. Moreover, this policy instrument has been widely criticized for being inflexible, complex and for not taking into consideration differences in technology and geography [102].

On the other hand, a carbon tax is simpler to design, has relatively low administration costs and is attractive to stakeholders in the building sector due to their familiarity with the tax mechanism. The revenues earned from carbon tax can be redistributed to other policy instruments such as incentives [7]. However, establishing an appropriate tax rate can be a challenging task for governments as it involves complete knowledge of costs of mitigation, the growth of the economy, progresses in technology and other factors which need to be taken into consideration. Moreover, due to opposition from the public and also to avoid pressuring the construction industry intensively, governments could also face problems in establishing a deterring tax rate that can reduce GHG emissions [102].

The cumulative amount of GHG emissions mitigated can be quantified with ETS and emission permits can be distributed for free or auctioned off. However, there are concerns of market failures and regulatory based loop holes because the construction sectors lacks proper GHG accounting [12]. It is necessary to move beyond the debate of policy instruments in order to be able to pinpoint the factors that are actually slowing the move to a carbon neutral construction industry. One of the common cited barriers to carbon reduction schemes in the construction industry is the incremental cost associated with it [103,104]. Studies have shown that building contractors and developers often overestimate the cost associated with energy efficiency [105]. For example, in Germany, new buildings with very little heating requirements can be constructed with an extra cost of no more than 5–12%, while, in Northern China, a building project was able to achieve reductions of 65% in heating consumption with an extra cost of no more than 8% without compromising thermal comfort [103,106]. Therefore, correct estimations are important for cost estimations.

Other cited barriers to carbon reduction in construction industry were the skills and knowledge gap of not only the designers and contractors but also of the end users, i.e., the occupants of the buildings [104,107]. As a conclusion, each instrument has some limitations; therefore, a variety of economic, environmental, political, and social factors need to be taken into consideration [7]. Training and education should be emphasized as important ways to reduce GHG emissions in the construction industry by enabling behavioral changes within organizations. In this context, identifying sources of the carbon footprint at the different stages during construction and showing possible carbon reduction technologies/systems and techniques as summarized in Table 4 will be helpful for awareness and to fulfill the knowledge gap at the design stage from clients to designers and contractors.

## 7. Conclusions

GHG emissions mitigation can be achieved by indirect pricing such as regulations and direct pricing such as carbon tax and emission trading schemes (ETS). However, regulations can be inflexible, complex, and may not take into consideration differences in technology and geography. In addition, ETS can be complex because the construction sector lacks proper GHG accounting. Therefore, increasing the awareness, education, and incentives can lessen the carbon footprint of construction industry. Consequently, we aimed to increase awareness of the carbon footprint sources in construction and building operations during manufacturing, transportation, construction, operations/management, and end-of-life deconstruction. As a result, various carbon reduction techniques/systems were identified. It was found that mining and manufacturing of materials and chemicals contributed to high energy usage and 90% of the total CO_2_ emissions. Therefore,

- Testing different blends of cement with addition of alternative additives such as alkali-activated slag mortars or fly ash in concrete;
- Changing cement production methods;
- Addition of Sasobit or reclaimed asphalt pavement in asphalt mixtures;
- Recycling building wastes such as concrete aggregate and recycled asphalt in common construction materials;
- Conversion from the wet process to the dry process in concrete manufacturing;
- Substitution of lower carbon content fuels for coal, coke, and petroleum coke;
- Alternate options in terms of vehicle type, engine power, truck capacity, and fuel type to improve the fuel efficiency in the construction vehicles;
- Reducing idle time by using direct fired heaters, auxiliary power units (APU), thermal storage systems, on-board batteries, and automatic engine shut-off devices;
- Applications of alternate water resources for water reuse purposes;
- Switching to efficient HVAC systems; and
- Utilization of different building operations/systems will lessen energy consumption and reduce GHG emissions up to 90% in different stages in construction industry.

This review can be useful at the stage of conceptualization, design, and construction to assist clients and stakeholders in selecting materials and systems. There is large scope for further research on how to decrease carbon footprint in construction. Some of the areas that require attention include:

- improving recyclable waste materials such as glass, rubber crumbs, etc., as construction materials;
- developing decision making tools for effective carbon footprinting;
- creating inventory databases for Life Cycle Assessment for each alternative material’s embodied carbon value.

## Figures and Tables

**Table 1:** On-site energy consumption and CO_2_ emissions from cement/concrete production annually. Adapted from [24,26].

Activity		Cement		Concrete	
		Energy Use/Ton (Btu)	CO_2_ Emissions (Ton)/Ton of Material	Energy Use/Ton (Btu)	CO_2_ Emissions (Ton)/Ton of Material
Quarrying and crushing		4.29 × 10^4^	4.05 × 10^−3^	1.61 × 10^5^	1.44 × 10^−2^
Cement manufacturing	Raw grinding	9.39 × 10^4^	1.69 × 10^−2^		
	Kiln fuels	4.62 × 10^6^	4.33 × 10^−1^		
	Reactions		5.44 × 10^−1^		
	Finish milling	2.71 × 10^5^	4.86 × 10^−2^		
Concrete production	Blending/mixing			3.54 × 10^5^	6.36 × 10^−2^
	Transportation			6.97 × 10^5^	5.10 × 10^−2^

**Table 2:** The energy type and consumption and the CO_2_ emissions associated with steel production. Adapted from [34].

Activity	Energy Type and Consumption	Final Energy (MBtu/Ton)	CO_2_ Emission (Ton)/Ton of Material
Primary steel making			
Sinter making	26 PJ fuel and 2 PJ electricity	0.264	0.009
Coke making	74 PJ fuel and 2 PJ electricity	0.718	0.007
Iron making	676 PJ fuel and 4 PJ electricity	6.421	0.120
Steel making (Basic oxygen furnace)	19 PJ fuel and 6 PJ electricity	0.236	0.005
Casting	15 PJ fuel and 11 PJ electricity	0.236	0.010
Hot rolling	157 PJ fuel and 34 PJ electricity	1.803	0.041
Cold rolling and finishing	43 PJ fuel and 15 PJ electricity	0.548	0.014
Boilers	167 PJ fuel and 0 electricity	1.577	0.085
Co-generation (integrated steel making)	101 PJ fuel and -22 PJ electricity	0.746	0.004
Secondary steel making			
Steel making using electric arc furnace	6 PJ fuel and 62 PJ electricity	0.642	0.031
Casting	1 PJ fuel and 4 PJ electricity	0.028	0.002
Hot rolling	102 PJ fuel and 22 PJ electricity	1.171	0.026
Cold rolling and finishing	Not required	none	-
Boilers	42 PJ fuel and 0 PJ electricity	0.397	0.026
Co-generation	11 PJ fuel and -2 PJ electricity	0.085	0.0004

**Table 3:** Reduction in CO_2_ emissions from alternative technology/materials in cement production. Adapted from [42].

Technology/Material	Alternative	Reduction in CO_2_ Emissions
Cement production methods	Fluidized bed kiln; high activation grinding	20 to 30 kg CO_2_/ ton product
Changes in raw material	Calcareous oil shale, steel slag	60 kg CO_2_/ton of clinker
	Carbide slag	374 kg CO_2_/ton of clinker
Emerging alternative cement products	Novacem cement	750 kg CO_2_/ton product
	Geopolymer cement	300 kg CO_2_/ton product
Carbon capture technologies	Calera cement manufacturing	500 kg CO_2_/ton of product
	Concrete curing	120 kg CO_2_/ton product
	Carbonate looping	370 to 500 kg CO_2_/ ton product
Fuel technologies	Oxygen enrichment and Oxy-fuel	404 to 676 kg CO_2_/ton cement
Post-combustion carbon capture	Absorption	690 to 725 CO_2_/ton clinker
Industrial recycling	CO_2_ from cement process into high-energy algal biomass	1800 kg of CO_2_ will be utilized per ton of dry algal biomass produced

**Table 4:** Summary of findings.

Building Operations	CO_2_ Emission	Reduction Material/Techniques	CO_2_ Reduction	References
Limestone quarrying	3.13 kg CO_2_-e per ton crushed rock product	Application of alternative/ renewable energy such as solar thermal and biodiesel as compared to acquiring energy needs for quarrying from the grid or natural gas	More than 81% reduction in GHG emissions annually	[24]
Portland clinker manufacturing	nearly 1 kg of CO_2_ per one kg of Portland clinker ^(b)^	Alternative clinker substitution—use of calcium carbide residue in replacement of limestone partially	374 kg CO_2_/ton of clinker annually, or more than 37% reduction in CO_2_ emissions per ton of clinker	[26,39]
Asphalt	0.05 ppm of CO_2_ per ton per year for conventional asphalt production	Sasobit additives with Warm Mix Asphalt	0.003 ppm to 0.004 ppm of CO_2_ per ton or more than 94% reduction in CO_2_ emissions	[38,51]
		Sasobit additives with Hot Mix Asphalt	0.005 ppm to 0.0054 ppm of CO_2_/ton, or more than 90% reduction in CO_2_ emissions	[38]
	132 kg CO_2_ equivalent /ton of virgin asphalt produced	Reclaimed asphalt pavement	Dropped to average of 84.35 kg CO_2_ equivalent/ton, or more than 36% reduction in CO_2_ emissions	[3,56]
Concrete	5 w/c were between 347 and 351 kg of CO_2_-e/m^3^	Recycled coarse aggregates	Reduce 0.03 tons of CO_2_-e/m^3^	[56,58]
	293 kg of CO_2_-e/m^3^	Fractional replacement of cement in concrete with fly-ash and ground granulated blast furnace slag and natural aggregates with recycle crushed aggregate	Reductions of up to 3.8% (10.5 kg CO_2_-e/m^3^)	[47]
Onsite construction process	(a) During idling, at a fuel consumption rate of 0.84 gal/hour, 2.7 kg CO_2_/liter was produced	Reducing idling time by using direct fired heaters instead of diesel engines	Direct fired heaters can reduce NOx and CO_2_ emissions by 99% and 94–96% respectively during idling time	[70]
	Traditional building with steel products or concrete produces 366 kg CO_2_-e/m^2^ total embodied carbon emissions	Using prefabricated wood instead of steel or concrete	25% reduction in total GHG emissions	[47,64]
	3-bedroom semi-detached house constructed using traditional masonry construction produces 405 kg CO_2_/m^2^	using offsite panelized timber frame and modern methods of construction	34% reduction in total embodied carbon emissions	[61]
Construction, demolition waste	0.004 to 0.01 kg CO_2_ per kg of the demolition waste	Recycling building demolition waste such as aluminum	20.07 kg CO_2_-e per kg of aluminum recycled	[26,78]
		Recycling waste steel from hoarding construction as steel scraps	281 kg CO_2_-e per m, or about 8% reduction in CO_2_ emissions	[64]
		Reusing wood waste into production-use of particleboard	14.6% reduction in CO_2_ emissions (−28.6 kg CO_2_-e/m^2^)	[47]
Building’s operations when in service	Account for 85.4% of the total emissions of a building’s life cycle	High energy performance air-conditioner	19 kg CO_2_-eq/m^2^	[47,48,82]
		Utilization of renewable energy such as a solar photovoltaic system, solar thermal water system, and a ground source heat pump	4.6 kg CO_2_-eq/m^2^	
		Use of natural ventilation and lighting	9.1 kg CO_2_-eq/m^2^	
		Use of sheep’s wool as insulation material in buildings	98% reduction in GHG emissions	
		Applying large thermal mass, concrete, and other heavy weight materials for reduction of HVAC energy	50% reduction in CO_2_ emissions/m^2^	[26]
		Rainwater harvesting system	8.5% reduction of GHG	[91]

## Data Availability

Not applicable.
